# Mapping the Physiological Response of *Oenococcus oeni* to Ethanol Stress Using an Extended Genome-Scale Metabolic Model

**DOI:** 10.3389/fmicb.2018.00291

**Published:** 2018-03-01

**Authors:** Angela Contreras, Magdalena Ribbeck, Guillermo D. Gutiérrez, Pablo M. Cañon, Sebastián N. Mendoza, Eduardo Agosin

**Affiliations:** ^1^Department of Chemical and Bioprocess Engineering, School of Engineering, Pontificia Universidad Católica de Chile, Santiago, Chile; ^2^Mathomics, Center for Mathematical Modeling, Universidad de Chile, Santiago, Chile; ^3^Center for Genome Regulation, Universidad de Chile, Santiago, Chile

**Keywords:** physiological ethanol response, wine-like defined culture medium, malolactic fermentation, lactic acid bacteria, *Oenococcus oeni*, genome-scale metabolic model

## Abstract

The effect of ethanol on the metabolism of *Oenococcus oeni*, the bacterium responsible for the malolactic fermentation (MLF) of wine, is still scarcely understood. Here, we characterized the global metabolic response in *O. oeni* PSU-1 to increasing ethanol contents, ranging from 0 to 12% (v/v). We first optimized a wine-like, defined culture medium, MaxOeno, to allow sufficient bacterial growth to be able to quantitate different metabolites in batch cultures of *O. oeni*. Then, taking advantage of the recently reconstructed genome-scale metabolic model iSM454 for *O. oeni* PSU-1 and the resulting experimental data, we determined the redistribution of intracellular metabolic fluxes, under the different ethanol conditions. Four growth phases were clearly identified during the batch cultivation of *O. oeni* PSU-1 strain, according to the temporal consumption of malic and citric acids, sugar and amino acids uptake, and biosynthesis rates of metabolic products – biomass, erythritol, mannitol and acetic acid, among others. We showed that, under increasing ethanol conditions, *O. oeni* favors anabolic reactions related with cell maintenance, as the requirements of NAD(P)^+^ and ATP increased with ethanol content. Specifically, cultures containing 9 and 12% ethanol required 10 and 17 times more NGAM (non-growth associated maintenance ATP) during phase I, respectively, than cultures without ethanol. MLF and citric acid consumption are vital at high ethanol concentrations, as they are the main source for proton extrusion, allowing higher ATP production by F_0_F_1_-ATPase, the main route of ATP synthesis under these conditions. Mannitol and erythritol synthesis are the main sources of NAD(P)^+^, countervailing for 51–57% of its usage, as predicted by the model. Finally, cysteine shows the fastest specific consumption rate among the amino acids, confirming its key role for bacterial survival under ethanol stress. As a whole, this study provides a global insight into how ethanol content exerts a differential physiological response in *O. oeni* PSU-1 strain. It will help to design better strategies of nutrient addition to achieve a successful MLF of wine.

## Introduction

The winemaking of red wines, and of some white wines, involves two fermentation processes: alcoholic fermentation conducted by yeast, and malolactic fermentation (MLF) performed by lactic acid bacteria (LAB). MLF allows wine deacidification and improves flavor complexity and microbiological stability (Henick-Kling et al., 1994; Zoecklein et al., 1999). This process consists on the decarboxylation of L-malate into L-lactate, a reaction that decreases wine acidity. *Oenococcus oeni* is the main bacterial species that carries out the MLF, due to its ability to grow under the harsh conditions present in wine, such as high ethanol content (>13% v/v), low pH (<3.5), and high sulphite concentration (<50 ppm) (Bauer and Dicks, 2004; Bartowsky, 2005; Li et al., 2006; Zapparoli et al., 2009; Zhang, 2013). However, *O. oeni* is not always able to achieve this task under these hostile conditions, often generating sluggish or stuck MLFs. For this reason, this process is considered one of the most difficult to manage during winemaking. Several studies have been carried out with the aim of understanding the metabolism of *O. oeni* under winemaking conditions; however, MLF still remains mostly unpredictable (Carreté et al., 2002; Bourdineaud et al., 2003; Da Silveira et al., 2003; Grandvalet et al., 2005, 2008; Olguín et al., 2009; Cafaro et al., 2014).

Survival of microorganisms under stress conditions requires the maintenance of the main functions of the cell membrane, which is essential to control ion permeability and to regulate solute exchange between the cell and the external medium. In wine, ethanol is considered the main stressor, because it can injure cell membrane integrity and impact cell viability.

Most studies on *O. oeni* survival in wine have focused on the deleterious effect of ethanol on the integrity of cell membrane and changes in the cell wall composition. Chu-Ky et al. (2005) reported that increasing ethanol content in the culture medium – from 10 to 14% v/v – increased membrane fluidity, resulting in significant loss of cell viability, even after 30 min of cultivation. Da Silveira et al. (2004) noticed an increase in the level of proteins involved in cell wall biosynthesis. Dols-Lafargue et al. (2008) showed that *O. oeni* strains able to express a functional *gtf* gene – involved in exopolysaccharide synthesis - were more resistant to ethanol and other stressors. Other authors determined that cells grown in the presence of 8% v/v ethanol modified the membrane fatty acid profile, resulting in an increment of membrane cyclopropane fatty acids (CFAs) (Teixeira et al., 2002; Grandvalet et al., 2008). The biosynthesis of CFAs from unsaturated fatty acids is catalyzed by a CFA synthase, encoded by the *cfa* gene. The correlation between *cfa* induction and ethanol resistance has been demonstrated in *O. oeni* cells (Grandvalet et al., 2008).

Nevertheless, the central metabolism of *O. oeni* under winemaking conditions is still scarcely understood. Indeed, several related studies did not include ethanol in the culture medium (Fourcassie et al., 1992; Salou et al., 1994; Maicas et al., 1999, 2002; Mohedano et al., 2014). Others, although including ethanol, have employed complex culture media, with the aim of satisfying increasingly demanding nutritional requirements of this bacterium with increasing ethanol content (Tracey and van Rooyen, 1988; Capucho and San Romao, 1994; Arena and Manca de Nadra, 2005; Olguín et al., 2009; Olguin, 2010; Bravo-Ferrada et al., 2016).

Complex media contain plant and/or animal water-soluble extracts and sugars which result in solutions rich in minerals and organic nutrients, but where the exact composition is unknown. On the contrary, defined culture media are composed of well-known chemical compounds in previously determined concentrations. The latter are, therefore, best suited than the former to fully assess the metabolic responses of a microorganism under different perturbation conditions.

To the best of our knowledge, an optimized, defined culture medium is not available yet to allow a quantitative characterization of the growth and metabolism of *O. oeni* under wine-like culture conditions. The only defined medium currently available for *O. oeni* has been reported by Terrade and Mira de Orduña (2009). This is a non-selective, chemically defined medium for *O. oeni*, as well as for other LAB, that provides strong growth comparable to standard laboratory media used for LAB like the MRS medium (De Man et al., 1960). However, Terrade′s medium does not include enological components, like malate, citrate, fructose or ethanol, among others.

Since the genome sequence of *O. oeni* PSU-1 was released by Mills et al. (2005), several -omic studies have significantly contributed to the understanding of the metabolic changes that occur in this microorganism during the MLF (Olguín et al., 2009, 2015; Olguin, 2010; Bordas et al., 2015; Costantini et al., 2015; Margalef-Catalá et al., 2016; Bartowsky, 2017; Sternes et al., 2017). Bridier et al. (2010) constructed a phylogenetic tree comparing the sequences of the seven housekeeping genes presents in 258 *O. oeni* strains. Then, two major phylogenetic groups were observed. Moreover, a third putative group was proposed comprising one strain, which was isolated from cider. Likewise, Campbell-Sills et al. (2015) revised the population structure of 50 *O. oeni* strains, using comparative genomics and confirmed that it can be divided in two major groups, according to their ecological niche, wine or cider. In congruence with Bridier’s work, a third group was proposed. Sternes and Borneman (2016) compared consensus pan-genome assemblies of the invariant (core) and variable (flexible) regions of 191 *O. oeni* strains. Genetic variation in amino acid biosynthesis and sugar transport and utilization was found to be common between strains. Moreover, other studies showed that *O. oeni* strains differed mainly in carbohydrate metabolism (Cibrario et al., 2016) and exopolysaccharide synthesis (Dimopoulou et al., 2014).

Besides, several studies have grouped *O. oeni* strains according to their capacity to perform the MLF or flavors production. Bon et al. (2009) observed the presence of eight stress-responsive genes in *O. oeni* strains that performed MLF more efficiently. El Khoury et al. (2017) reported the isolation, genotyping, and geographic distribution analysis of 514 *O. oeni* strains. Their phylogenetic relationships were evaluated using a method based on single nucleotide polymorphism (SNP) analysis. The results show that strains are not genetically adapted to regions but to specific types of wines. More recently, Sternes et al. (2017) found thirteen genes differentially expressed in the strains analyzed, which were associated with the production of diacetyl, a commercially valuable aroma compound. Finally, Campbell-Sills et al. (2017), studying the genomics and metabolomics of 14 *O. oeni* strains isolated from Burgundy, identified two different *O. oeni* lineages associated to either red or white wines in this French region.

Transcriptomic and proteomic analyses of *O. oeni* strains cultivated in wine-simulated cultures showed that the environment strongly affects *O. oeni* stress responses at this level. Costantini et al. (2015) found that under mild ethanol stress culture conditions, (8% v/v), genes codifying for chaperones with refolding activity were over-expressed; and at higher alcohol concentration (12%v/v), genes that codify for chaperones with proteolytic activity were induced. Olguín et al. (2015) performed transcriptomic and proteomic analyses which revealed that main genes affected by ethanol (12%v/v) were related with metabolite transport, as well as cell wall and membrane biogenesis; furthermore, they observed relocation of cytosolic proteins in the membrane, as a protective mechanism. More recently, Margalef-Catalá et al. (2016) also performed transcriptomic and proteomic analyses, showing that the amino acid metabolism and transport were altered and that several peptidases were up regulated both at gene and protein levels. More recently, Margalef-Catalá et al. (2016) also performed transcriptomic and proteomic analyses, showing that the amino acid metabolism and transport mechanisms were altered and that several peptidases were up-regulated both at gene and protein levels. Moreover, the authors observed that genes related with malate and citrate uptake were up-regulated, while genes related with fructose consumption were down-regulated.

Otherwise, over the last 16 years, more than 80 genome-scale metabolic models (GSMM) have been reconstructed, which has been of substantial help in the study and applications of corresponding biological systems. Initially, GSMM have considered only highly characterized organisms, such as *Escherichia coli* and *Saccharomyces cerevisiae*; since then, its use has been expanded to other, less characterized species, as well as to complex biological systems. Nowadays, genome-scale models are widely used for studying the metabolism of numerous organisms, including LAB such as *Lactococcus lactis* (Oliveira et al., 2005; Verouden et al., 2009; Flahaut et al., 2013), *Lactobacillus plantarum* (Teusink et al., 2006), *Streptococcus thermophilus* (Pastink et al., 2009) and, more recently, Enterococcus *faecalis* (Veith et al., 2015) and *Streptococcus pyogenes* (Levering et al., 2016). These models have been employed for analyzing growth, auxotrophies and flavor formation; they have also assisted in the process of selection and development of strains with enhanced industrial utility. In a previous work carried out by our group, we developed the first genome-scale metabolic model for an *O. oeni strain* (Mendoza et al., 2017). We reported the general features of the model, as well as its predictive capabilities. The genome sequence of *O. oeni* PSU-1 strain was employed for this purpose.

In this work, we first designed a defined culture medium simulating wine conditions, able to support *O. oeni*’s growth at different levels of ethanol; then, we characterized the evolution of different nutrients and metabolic products during the fermentation. Finally, taking advantage of the recently constructed genome-scale metabolic model (GSMM) of *O. oeni* PSU-1 strain (named iSM454) (Mendoza et al., 2017), we predicted the metabolic behavior and the nutritional requirements of *O. oeni* at different growth phases, under increasing ethanol concentrations. The results clearly indicate that differential nutritional requirements of the *O. oeni* PSU-1 strain are required when ethanol concentration increases. As a whole, this work contributes to a better understanding of *O. oeni* metabolism under oenological conditions, as well as to the identification of essential nutrients required for survival of this bacterium in the different stages of growth.

## Materials and Methods

### Microorganisms and Media

*Oenococcus oeni* (Garvie, 1967; Dicks et al., 1995) (PSU-1, ATCC^®^ BAA-331^TM^) was obtained from the American Type Culture Collection (ATCC) (Manassas, VA, United States). Cryogenically preserved (-80°C) strains were cultured and maintained on MRS plates (Man, Rogosa, and Sharpe) (De Man et al., 1960) and stored at 4°C.

An *O. oeni* PSU-1 preculture was prepared from a frozen stock by inoculating 100 ml Erlenmeyer flasks containing 75 ml MRS medium supplemented with 0.5 g L^-1^ of cysteine. Before inoculation, the cells were subjected to ethanol adaptation. For this purpose, we serially passaged every culture, starting from 1% ethanol v/v to reach 0, 3, 6, 9, or 12% v/v ethanol concentration in each culture. *O. oeni*’s cells were inoculated in each culture medium to achieve an initial optical density at 600 nm (OD_600_) of approximately 0.1. When the cultures reached OD_600_ = 0.2, they were transferred to other cultures containing higher ethanol content.

### Design of a Chemically Defined Culture Medium

The developed chemically defined culture medium, named MaxOeno, was designed with the aim of simulating the wine environment, allowing *O. oeni* to grow in the presence of ethanol. However, the concentration of carbon and nitrogen used in MaxOeno was higher than that found in wine, because these concentrations allowed the growth of *O. oeni* avoiding its arrest or slowing. The vitamins and minerals were those of Terrade and Mira de Orduña (2009), but their concentration was increased three fold. Using literature data, we verified that these concentrations were not inhibitory for the bacterium growth (Mesas et al., 2004).

Carbon and energy sources were those normally found in wine, (i.e.) glucose, fructose, malate and citrate. Glucose and fructose are the main residual sugars present in wine. Both were added in equal concentrations (12.5 g L^-1^) with the aim of studying their metabolic fate in *O. oeni*. Meanwhile, malate and citrate, the main organic acids in wine, were included at a concentration of 5 and 1 g L^-1^, respectively. We verified from the literature that these concentrations were not inhibitory for the bacterium, and that they also allow bacterial cells to grow in the presence of ethanol (Salou et al., 1991; Saguir and Manca de Nadra, 1996; Maicas et al., 2000; Mesas et al., 2004; Augagneur et al., 2007; Olguin, 2010).

The amino acids content was calculated using their yields in biomass. For this purpose, we employed the yields reported for *Lactobacillus plantarum* and *Lactococcus lactis* (Novak et al., 1997; Teusink et al., 2006), because we did not find any reported data for amino acid yields in *O. oeni*.

#### Composition of the MaxOeno Culture Medium

The MaxOeno culture medium contained, in g L^-1^: citrate 1, malate 5, calcium chloride (dihydrate) 0.4, magnesium sulfate 1.3, fructose 12.5, glucose 12.5, dipotassium phosphate 2.0. Tween 80 was also added at 1 ml L^-1^, as well as a nitrogenous bases solution, 100 mL L^-1^; a mineral salts solution, 5 ml L^-1^; and a vitamins solution, 1 ml L^-1^.

The vitamin solution contained the following, in g L^-1^: thiamine, 1; biotin, 1; nicotinic acid, 2; pyridoxine hydrochloride, 2; C-D-pantothenate, 2; folic acid, 1; choline chloride, 2; riboflavin, 1; 4-aminobenzoic acid, 0.1; cyanocobalamine, 0.1; and xanthine, 5. The nitrogenous bases solution contained: adenine sulfate, 0.5; uracil, 0.5; cytosine, 0.5; thymine, 0.5 and guanine, 0.5. Mineral salts solution contained: MgSO_4_^∗^7H_2_O, 60; FeSO_4_^∗^7H_2_O, 12; CuSO_4_^∗^5H_2_O, 0.015; and ZnSO_4_^∗^7H_2_O, 0.135. The vitamin solution was sterilized by membrane filtration (pore size < 0.22 μm, Millipore, United States).

Besides, the culture medium was supplemented with 1,060 mg L^-1^ of assimilable nitrogen prepared with the following amino acids, in g L^-1^: L-arginine 0.4, L-serine 0.24, L-threonine 0.27, L-glutamic acid 0.33, L-aspartic acid 0.3, L-lysine 0.33, L-asparagine 0.3, L-leucine 0.30, L-glutamine 0.50, L-alanine 0.2, cysteine 0.54, glycine 0.27, histidine 0.53, isoleucine 0.30, methionine 0.34, phenylalanine 0.37, proline 0.67, tryptophan 0.46, tyrosine 0.41 and valine 0.27.

Before sterilization, the pH of the medium was adjusted to 4.8 using KOH.

### *O. oeni* Cultivation in Different Ethanol Conditions

Ethanol-adapted cells were inoculated in 100 mL flasks containing 70 mL of MaxOeno culture medium to achieve an initial optical density at 600 nm (OD600) of approximately 0.2. The flasks were of glass, airtight and with a sampling port. The cultures were incubated at 25°C, without stirring. Samples were collected aseptically through the flask sampling port, and bacterial growth was estimated by OD600, the optical density of the culture measured at 600 nm. At the same time, the content from each flask was centrifuged; the supernatant was collected and frozen at -20°C, for future chemical analyses.

The biomass was determined as dry weight of cells through a calibration curve of OD_600_ versus dry weight (g L^-1^). The latter was previously carried out as described in Li and Mira de Orduña, 2010 to obtain equation (1), where both parameters are related.

$$X(g\,\;DCWL^{-1})=0.8105\times ({OD}_{600})+0.0104$$

### Chemical Analyses

L-lactate, D-lactate and amino acids were quantified by UHPLC/MS using a Dionex unit model Ultimate-3000 (Dionex Corp., Sunnyvale, CA, United States) coupled to a mass spectrometer Exactive^TM^ plus (Thermo Fisher Scientific, San Jose, CA, United States). The UHPLC system was controlled using the Xcalibur^TM^ 2.13 software (Thermo Fisher Scientific, San Jose, CA, United States). The methods utilized for compound identification and quantification are detailed below:

#### L- and D-lactate

Fifty microliter of sample were dried and then derivatized by adding 50 μl of (+)-*O,O′*-diacetyl-L-tartaric anhydride solution (≥97%) (DATAN) (Sigma–Aldrich, United States) [100 mg ml^-1^, where DATAN was dissolved in dichloromethane:acetic acid (4: 1, by volume)]. The samples were incubated for 40 min at 75°C, under agitation. Subsequently, the samples were dried and reconstituted in 200 μl of a solution of acetonitrile and water (1:2). L-lactate and D-lactate (≥98%) (Sigma–Aldrich, United States) were used as external standards. A 10 μl derivatized sample was injected in the equipment and separated using a UPLC BEH C18 (100 × 2.1 mm, 1.7 μm, Waters) analytical column at a flow of 0.5 ml min^-1^ and oven temperature of 31°C. Solvents used for separation were: solvent A: ammonium formate 1.5 mM, pH was adjusted at pH 3.6 using formic acid. Solvent B: acetonitrile.

#### Amino Acids

A 10 μL sample was directly injected in the equipment and separated using a LiChrospher^®^ 100 RP-18 (5 μm) (Merck) analytical column with a flow of 0.35 ml min^-1^ and oven temperature of 30°C. Solvents used for separation were: solvent A: formic acid 0.1% v/v. Solvent B: methanol.

#### Cysteine

A 50 μl sample was derivatized using 100 μl 5,5′-dithiobis(2-nitrobenzoic acid) (Ellman’s reagent) (Sigma–Aldrich, United States). The reagent solution for derivatization was prepared using 4 mg Ellman’s reagent dissolved in 10 ml buffer phosphate 0.01 M (pH 7.0). All samples were derivatized at the time of being taken. The chromatographic conditions were the same of those employed for amino acids analysis.

Sugars (glucose, fructose), organic acids (malate, acetate, citrate, total (L- + D-) lactate and alcohols (ethanol, mannitol, and erythritol) were separated and quantified in a Lachrom L-700 HPLC system (Merck Hitachi, Japan) equipped with Diode Array and Refractive Index detectors (Hitachi, Japan). An Aminex HPX-87H ion exchange column (Bio-Rad, United States) was used, as described previously (Varela et al., 2003). For s*ugars, malate, citrate, acetate, and ethanol*, the mobile phase used was sulphuric acid 5 mM with a flow of 0.450 ml min^-1^ and oven at 35°C. For *mannitol and erythritol*, the mobile phase was milliQ^TM^ water with a flow rate of 0.450 ml min^-1^ at a constant temperature of 75°C. External standards were used to quantify the required compounds in all cases.

### Genome-Scale Metabolic Model

**FIGURE 1:**
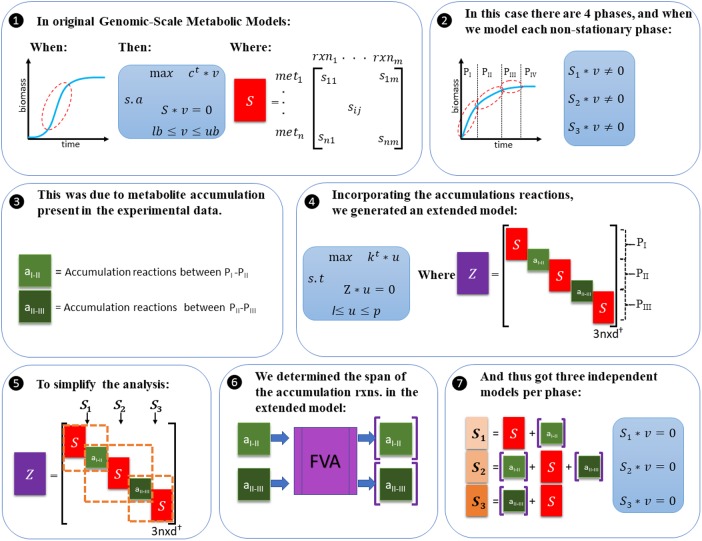
Framework for the incorporation of the experimental data. (1) General approach used to model exponential phase in GEMS. (2) Our experimental results showed four phases: three growth phases and a fourth phase, corresponding to stationary phase. Growth phases couldn’t be modeled individually as there was accumulation, as observed in the experimental results. (3) These accumulation compounds were: malate and mannitol, and the amino acids valine, phenylalanine, cysteine and threonine. Accumulation was observed between phases I to II, and II to III, and not from III to IV. (4) The extended model was constructed, which was able to simulate simultaneously the three phases identified for each ethanol level, and the accumulation observed experimentally. This matrix includes the following components: a matrix “Z” that contains three “S” matrix, one per phase, and 2 “a” vectors (1 × 6), where “a” represents the accumulation reactions that allows interaction between phases; thus, matrix sigma has a size (3n × d), where *d* = 3m + 2^∗^6. The extended model also includes vector “u” (1 × d) of internal fluxes, limited by vector “l” and “p” (1 × d), and a vector “k” of weights (1 × 3n). (5) To simplify the analysis, the extended matrix can be divided into three independent problems, one corresponding to each phase, and thus each one includes an S matrix, and a set of accumulation reactions as input of the system (accumulation in the previous phase) and/or output of the system (accumulation in the consecutive phase). (6) The span of these accumulation reactions was determined with flux variability analysis in the extended model, with the experimental data fixed in the model. (7) Estimations and further calculations were carried out in the independent model described in (5), with the span determined in (6) for the accumulation reactions.

Genome-scale metabolic models can be used to find flux distributions under the assumption of steady state. In steady state, the concentration of intracellular metabolites remains constant and all the mass produced must be consumed in order to fulfill the mass balances. No accumulation of intracellular metabolites is allowed. GSMMs can also be used to model the exponential phase (**Figure 1**, step 1) where a pseudo steady state is accomplished. Thus, we modeled each of the three differentiated growth phases observed during growth of *O. oeni* PSU-1 (**Figure 1**, step 2). However, experimental results suggested that intracellular accumulation of some metabolites occurred during growth, and thus the assumption of no accumulation could not be applied in this case (**Figure 1**, step 3).

In the most widely used approach, Flux Balance Analysis, a matrix S, which summarizes the biochemical reactions occurring in a metabolic network, is used. In this matrix, the stoichiometric coefficients are used to describe each reaction. The assumption of steady state indicates that mass balances must be accomplished, which is equal to state S^∗^v = 0, where v is a vector of reaction fluxes. As we observed experimental accumulation of metabolites, we decided to include that accumulation in the form of sink and demand reactions. In particular, accumulation reactions were added in the form of sink reactions for phases II and III, and demand reactions for phases I and II (**Figure 1**, step 5). To determine the variation range of these accumulation reactions, an extended model was built, able to simulate simultaneously the three growth phases, in which the assumption of S^∗^v = 0 was still valid (**Figure 1**, step 4). This extended model was employed to calculate the maximum and minimum flux that these accumulation reactions were able to carry under the restrictions fixed by the experimental results. Then, these constraints were included in the iSM454 model and used for further analysis.

The GSMM of *Oenococcus oeni* PSU-1 recently developed by our group was employed in this work (Mendoza et al., 2017). GSMM allows to model the exponential phase of growth curves (**Figure 1**, step 1); thus, this approach was used here for modeling each of the four differentiated growth phases (three growth phases + stationary phase) observed during growth of *O. oeni* PSU-1 (**Figure 1**, steps 2). However, experimental results suggested that intracellular accumulation of some metabolites occurred during growth, and thus the assumption of no accumulation, as well as the restriction S^∗^v = 0, could not be applied in this case (**Figure 1**, step 3), unless the accumulation was represented in the model. Therefore, to include the latter in the S matrix, accumulation reactions were added in the form of sink reactions for phases II and III, and demand reactions for phases I and II (**Figure 1**, step 5). To determine the variation range of these reactions, an extended model was built, able to simulate simultaneously the three growth phases, in which the assumption of S^∗^v = 0 was still valid (**Figure 1**, step 4). This extended model was employed to calculate the maximum and minimum flux that these accumulation reactions were able to carry under the restrictions fixed by the experimental results. Then, these constraints were included in the iSM454 model and used for further analysis.

#### Construction of the Extended Model

An extended model was generated to allow simultaneous simulation of the three phases observed for each ethanol level (**Figure 1**, step 4). This model was used to determine the maximum and minimum fluxes required through the accumulation reactions so that each independent phase simulation was able to fulfill the S^∗^v = 0 assumption. This extended model possesses a stoichiometric matrix Z, which contains three “S” sub matrices (**Figure 1**, step 4), each associated to a specific growth phase. These “S” sub matrices were taken from iSM454 and represent three independent problems solved simultaneously in the extended model. Then, to allow interaction between these three problems and thus lose independency, accumulation reactions were added, which allow flux to pass from phase_n_ to phase_n+1_. These were selected based on experimental results, and corresponded to the following compounds: mannitol, malate, cysteine, threonine, phenylalanine, and valine.

These reactions had the following structure:

mannitol_cytosol1→mannitol_cytosol2

where *mannitol _cytosol1* represents cytosolic mannitol in growth phase I, and *mannitol _cytosol2* represents corresponding cytosolic mannitol in growth phase II.

#### Determination of the Constraints for the Accumulation Reactions

To determine the maximum and minimum values that the accumulation reactions were able to carry under the restriction of the experimental constraints, the following fluxes (mmol gDCW^-1^ h^-1^) were set in the extended model, according to the corresponding phase, with experimental values: the substrates glucose, fructose, citrate, malate, cysteine, threonine, valine, phenylalanine and serine; and the products mannitol, erythritol, L-lactate, D-lactate and acetate (**Figure 1**, step 4). To minimize experimental error, linear regressions were determined for each of the metabolites at each growth phase and used to predict the phase’s final value. Initial values were taken from the initial concentration of the experiment for phase I, and from the final values of the former phase for phases II and III.

Then, flux variability analysis (FVA) was carried out to determine the maximum and the minimum flux that each accumulation reaction was able to carry under these constrained conditions.

#### Generation of the Three S’ Matrixes

When the ranges of the accumulation reactions were determined, the extended model was divided into three S’ matrix, each one representing one of the growth phases. These were used to carry out all the further analysis. Each of these matrixes include the S matrix from iSM454, and also a set of the accumulation reactions as sink reactions for phases II and III, and demand reactions for phases I and II (**Figure 1**, step 5), to represent the mass difference observed experimentally as an input or/and output of the system. From now on in the text, every time the iSM454 model is mentioned, it will refer to the model in which the S’ matrixes are included.

#### Flux Balance Analysis (FBA)

Flux balance analysis was carried out in the iSM454 model (with the S’ matrixes) to analyze each of the different growth phases at each ethanol level. In order to do so, the following fluxes (mmol gDCW^-1^ h^-1^) were set with experimental values: the substrates glucose, fructose, citrate, malate, cysteine, threonine, valine, phenylalanine and serine; and the products mannitol, erythritol, L-lactate, D-lactate and acetate. The accumulation reactions were constrained with the ranges determined with the extended model. Also, experimental biomass and NGAM estimated in this work were fixed.

#### Prediction of Non-growth Associated Maintenance (NGAM)

Non-growth associated maintenance was estimated by setting the specific production and consumption rates of the experimentally measured compounds, as described above for FBA. Thereby, flux through the NGAM reaction was progressively increased from 0 to 4 mmol gDCW^-1^ h^-1^. In each cycle, biomass production rate was maximized, and the prediction error was assessed. The NGAM flux that allowed the lowest biomass prediction error was selected.

#### Sensitivity Analysis for NGAM

Sensitivity of estimated NGAM was assessed for each of the specific consumption/production rates set in the model. For this purpose, each rate was varied independently by increasing and decreasing its value in 1%, and then NGAM was recalculated each time.

#### Determination of Energetic and Redox Requirements

The flux distribution obtained through FBA was used to quantify ATP, and NAD(P)^+^/NAD(P)H utilization. In *O. oeni*, ATP is produced through the F_0_F_1_-ATPase and/or through three reactions of the phosphoketolase pathway that involve the following enzymes: acetate kinase, pyruvate kinase and 3-phosphoglycerate kinase. To quantify the NAD(P)^+^/NAD(P)H utilization, the reactions that produce NADH and NADPH were analyzed, that correspond to reactions that involve the following enzymes: malate dehydrogenase, glyceraldehyde-3P dehydrogenase, threonine dehydrogenase, NADH quinone reductase, NAD(P)^+^ transhydrogenase and the pathway for methylglyoxal degradation for NADH formation; and Glucose-6P dehydrogenase, phosphogluconate dehydrogenase and GMP reductase for NADPH formation. This knowledge was considered for the determination of the maximum intracellular fluxes for NAD(P)^+^/NAD(P)H and ATP synthesis at different growth phases.

#### Flux Variability Analysis

Flux variability analysis was carried out by maximizing and minimizing the flux under the same constrained conditions used for FBA. This technique was applied to each of the accumulation reactions present in the extended model, and in iSM454 to analyze changes of production of ATP, NADH, NADPH; and separately, in F_0_F_1_-ATPase. To assess variation of ATP, NADH and NADPH, the sum of the reactions described above was subjected to FVA for each cofactor.

#### Elementary Flux Mode Analysis (EFMA)

As our culture medium mimics the wine composition, it contains different carbon sources and thus it is difficult to elucidate which substrate is being used to synthesize a particular product. Therefore, we used EFMA to find which metabolic products can be generated from each substrate, separately. EFMA was carried out with CellNetAnalyzer version 2017.4 (Klamt et al., 2007; Klamt and von Kamp, 2011; von Kamp et al., 2017). In order to do so, a reduced version of the iSM454 model was constructed. This model contained the central carbon metabolism of *O. oeni* PSU-1, that includes: phosphoketolase pathway, fructose reduction, citrate degradation and MLF. It also included energetic reactions such as F_0_F_1_-ATPase, NGAM, and the pathways for amino acid degradation present in *O. oeni* PSU-1, which correspond to serine, threonine and cysteine. A complete list of the reactions included is shown as **Supplementary File S1**.

### Random Sampling Analysis

To determine the flexibility of the metabolic network under ethanolic conditions, a Random Sampling Analysis was conducted. In this analysis, the solution space is uniformly sampled, and flux distributions can be explored to assess the flexibility of different pathways. In contrast to EFMA, random sampling can be conducted in the range of minutes to hours for large metabolic network resulting in a valuable tool to explore the solution space without requiring the use of a reduced model.

Flux distributions were calculated using optGpSampler (Megchelenbrink et al., 2014). For each condition, the network was first constrained using uptake and production rates as well as sink reactions associated with metabolites accumulation. Then, 100.000 flux distributions were calculated for each constrained metabolic network using standard parameters (number of parallel threads = 4, number of steps between samples = 500, solver = gurobi 7.52). The flexibility of the network in each condition was assessed through the comparison of flux variations for 40 reactions belonging to phosphoketolase pathway. Standard deviations among the previously calculated 100.000 flux distributions were computed for each of the 40 reactions. Higher standard deviations were considered as indicators of higher network flexibility.

## Results

### Cell Growth of *O. oeni* PSU-1 Strain in Defined Wine-Like Culture Medium

The ability of *O. oeni* to grow in the defined, wine -like culture medium designed here, named MaxOeno, was followed by biomass concentration (measured as optical density, OD_600nm_) and specific growth rates. Additionally, we compared the performance achieved in this medium with the defined medium previously described by Terrade and Mira de Orduña (2009). The growth in both media was evaluated either with (12% v/v of ethanol) and without ethanol; and at pH 3.5, in both cases. *O. oeni* PSU-1 cultivated in MaxOeno’s grew more (OD_600nm_ = 0.75 vs. OD_600nm_ = 0.25) and faster (μ = 0.012 h^-1^ vs. μ = 0.004 h^-1^) than Terrade’s. Therefore, the MaxOeno culture medium was employed for all further experiments of this work.

### Growth of *O. oeni* PSU-1 Strain Cultivated in the Presence of Increasing Ethanol Concentrations

**FIGURE 2:**
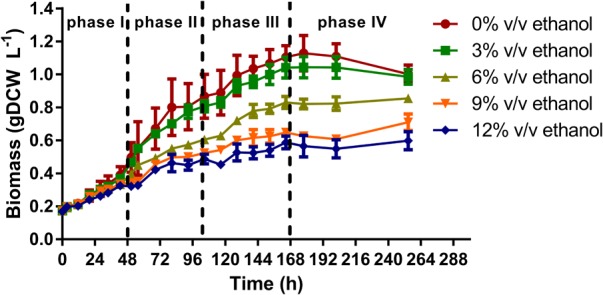
Effect of ethanol concentration on the growth of *O. oeni* PSU-1 strain cultivated in MaxOeno, a defined wine-like culture medium. Each growth phase is delimited by discontinued vertical lines. Phases I, II III, and IV last between 0 to 48 h, 48 to 104 h and 104 to168 h and 168 to 264 h of cultivation, respectively.

*Oenococcus oeni* PSU-1 was grown in MaxOeno culture medium with increasing ethanol concentrations (0, 3, 6, 9, or 12% v/v ethanol), at pH 4.8. Cell growth kinetics featured four growth phases for all culture conditions, (i.e.) phases I, II, III and stationary; ranging from 0 to 48 h, 48 to 104 h, 104 to 168 h and 168 to 264 h cultivation, respectively. These phases were characterized by different substrate consumption, e.g., during phase I, over 90% of malate and citrate were consumed; phase II presented a higher consumption rate of fructose and glucose, if compared to phase III; and phase III presented a slower specific growth rate, due to a strong decrease in sugar consumption rates (**Supplementary Figure S1**). It is worth mentioning that during growth, a lag phase was not observed in all cases (**Figure 2**). Besides, all cultures reached maximum biomass titers after 164 h (6.8 days) cultivation; thereafter, growth arrested.

A clear, linear relationship (*r*^2^ = 0.98) between ethanol concentration and cell growth was found, when considering each phase. Moreover, cultures with higher ethanol content showed slower specific growth rate and lower biomass production compared to cultures without ethanol (**Table 1**). The maximum biomass production and specific growth rate were observed during phase I.

**Table 1:** Maximum biomass production (gDCW L^-1^) and maximum specific growth rates (h^-1^) of *O. oeni* PSU-1 cultivated in MaxOeno defined medium with increasing concentrations of ethanol.

Ethanol content (% v/v)	Maximum biomass production^(¥)^ (gDCW L^-1^)	Maximum specific growth rate^(¥)^ (h^-1^)
0	0.854^1^	0.021^1^
3	0.76^2^	0.018^2^
6	0.585^3^	0.016^3^
9	0.452^4^	0.014^4^
12	0.447^4^	0.013^5^


*¥All assays were carried out in triplicate and its coefficients of variation (CV) were <20%. Shared superscript numbers in the same column indicate no significant difference between ethanol conditions (Mood test, *p* < 0.05).*

Stationary phase was not considered in further metabolic studies because neither growth, nor consumption or production of compounds was observed.

### Metabolism of Sugars and Organic acids by *O. oeni* PSU-1 Strain

*Oenococcus oeni* employs three metabolic pathways to obtain energy: phosphoketolase pathway (heterolactic fermentation), MLF and citrate degradation. The end-products of these catabolic pathways are D-lactate, L-lactate, acetate, ethanol, mannitol, erythritol and CO_2_. To assess the metabolic response of *O. oeni* to ethanol concentration, we followed the consumption and production of the major metabolites of these pathways.

#### Glucose and Fructose Consumption

**FIGURE 3:**
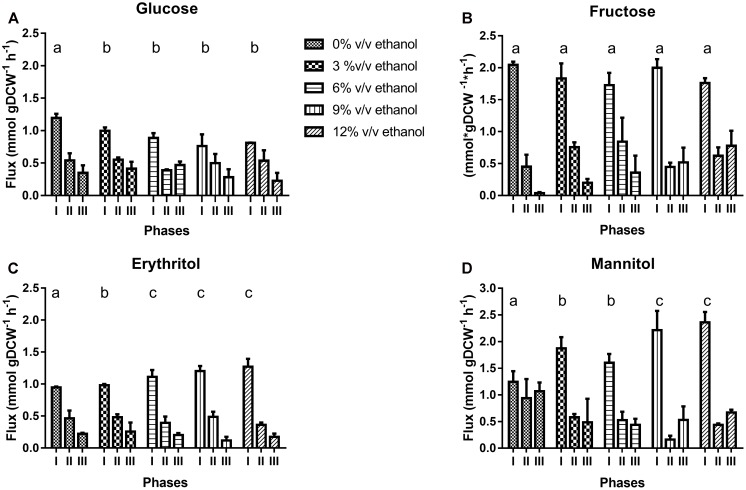
Specific consumption of glucose **(A)**, and fructose **(B)** and concomitant specific production of the related products, erythritol **(C)** and mannitol **(D)**. during cultivation of *O. oeni* PSU-1 under increasing ethanol contents. Statistical analysis only was performed in phase I and shared letters indicate no significant difference (Mood test, *p* < 0.05).

As expected, the higher the ethanol concentration in the medium, the lower the sugar consumption. However, whatever the ethanol concentration was, the largest amounts of sugars were consumed during phase I. In this phase, total sugar consumption was similar for all culture conditions (**Supplementary Figure S1**), although specific consumption rates for fructose were twofold faster than for glucose, independently of ethanol concentration present in the culture medium (**Figures 3A,B**).

During phase II, both glucose and fructose consumption increased when the ethanol concentration present in culture medium was lower (**Supplementary Figure S1**). Furthermore, during this phase, *O. oeni* PSU-1 consumed glucose faster than fructose. Specific consumption rate of fructose was reduced by 50% compared to the previous phase, although specific glucose consumption rate decreased less, regardless of culture conditions (**Figures 3A,B**).

Finally, in phase III, specific consumption rate of glucose was generally faster than fructose. Specific glucose consumption rates in cultures without and with 3 and 6% v/v ethanol were similar than phase II; but decreased significantly in cultures with 9 and 12% v/v ethanol (**Figures 3A,B**). Besides, specific fructose consumption rates in cultures without and with 3% v/v ethanol decreased around 90 and 60%, respectively. Similar rates than in phase II were found for cultures with 6, 9, and 12% ethanol.

#### Mannitol and Erythritol Production

These polyols are mainly produced through fructose catabolism - although they could also be synthesized from glucose. Again, the fastest production of mannitol and erythritol was achieved during phase I. The production of both metabolites increased concomitantly, with ethanol content (**Figures 3C,D**). Total mannitol concentration - the most abundant metabolite produced - was higher than erythritol concentration. In the absence of ethanol, specific production rates for mannitol and erythritol were 1.25 mmol gDCW^-1^h^-1^ and 0.95 mmol gDCW^-1^ h^-1^, respectively. Production rates of both polyols raised concomitantly with ethanol increment, during phase I (**Figures 3C,D**).

In phase II, production rates of both polyols dropped by half of the previous phase in the absence of ethanol in the culture medium. Once again, mannitol production increased with ethanol content, except for cultures with 6% ethanol (**Figure 3D**). On the contrary, erythritol production sharply decreased in this phase for cultures containing 9 and 12%v/v ethanol (**Figure 3C**).

#### L-malate and Citrate Metabolism

L-malate and citrate are the main organic acids in wine that support the growth of *O. oeni* under nutritional stress conditions. Lactate is synthesized by *O. oeni* as L- or D-enantiomers, according to its origin. L-lactate is produced in one step from L-malate through MLF; and D-lactate from the phosphoketolase pathway.

**FIGURE 4:**
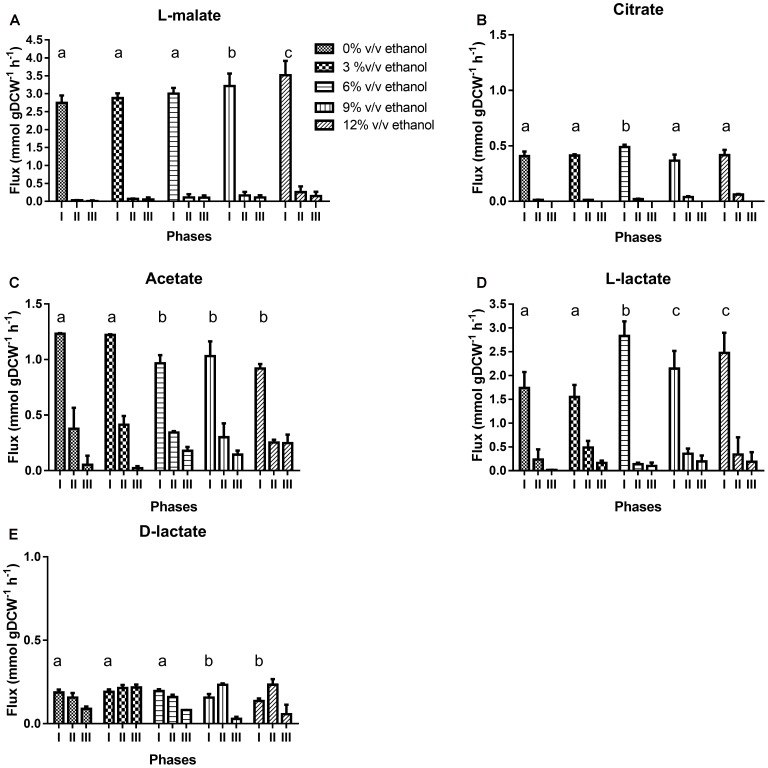
Specific consumption of L-malate **(A)** and citrate **(B)** and specific production of acetate **(C)** L-lactate **(D)** and D-lactate **(E)** during growth of *O. oeni* PSU-1, under increasing ethanol contents. Statistical analysis only was performed in phase I and shared letters indicate no significant difference (Mood test, *p* < 0.05).

L-malate and citrate were metabolized during phase I (**Figures 4A,B**), their consumption being triggered at the very beginning of the cultivation. Total acid consumption, expressed in concentration units (g L^-1^), suggests that all cultures behaved similarly, both acids being completely consumed at the end of phase I (i.e.) after 48 h cultivation (**Supplementary Figure S2**). In relation to specific consumption rates, L-malate was consumed faster when ethanol concentration was higher in culture medium; however, citrate was not differentially consumed.

Surprisingly, malate consumption was larger than L-lactate production; and the specific consumption rate of the former was *circa* 30% faster than the latter, for all culture conditions (**Figures 4A,D**). This suggests that one of these compounds is either accumulating intracellularly or being transformed into another metabolite (**Figures 4A,D**).

Otherwise, the specific production rate of D-lactate (0.15–0.20 mmol gDCW^-1^ h^-1^) was more than ten fold slower than the corresponding one for L-lactate (1.5–3.0 mmol gDCW^-1^ h^-1^), at least during phase I. Also, during this phase D-lactate production is lower at 9 and 12% ethanol content. Contrary to L-lactate, D-lactate is still significantly synthesized in later phases, although at variable rates (**Figures 4D,E**).

Acetate was the second most abundant metabolite produced, reaching a maximum of 50 mmol gDCW^-1^ in cultures without ethanol. *O. oeni* produces acetate from citrate and from sugars through the phosphoketolase pathway. During phase I, citrate uptake and acetate synthesis rates were higher than other phases (**Figures 4B,C**), suggesting that acetate was formed using all citrate consumed. However, acetate synthesis was faster than citrate uptake, which indicates that another pathway as phosphoketolase pathways was used additionally for its production. In later phases, however, acetate was mainly produced through phosphoketolase pathway, using other compounds, such as glucose.

### Metabolism of Amino Acids

**FIGURE 5:**
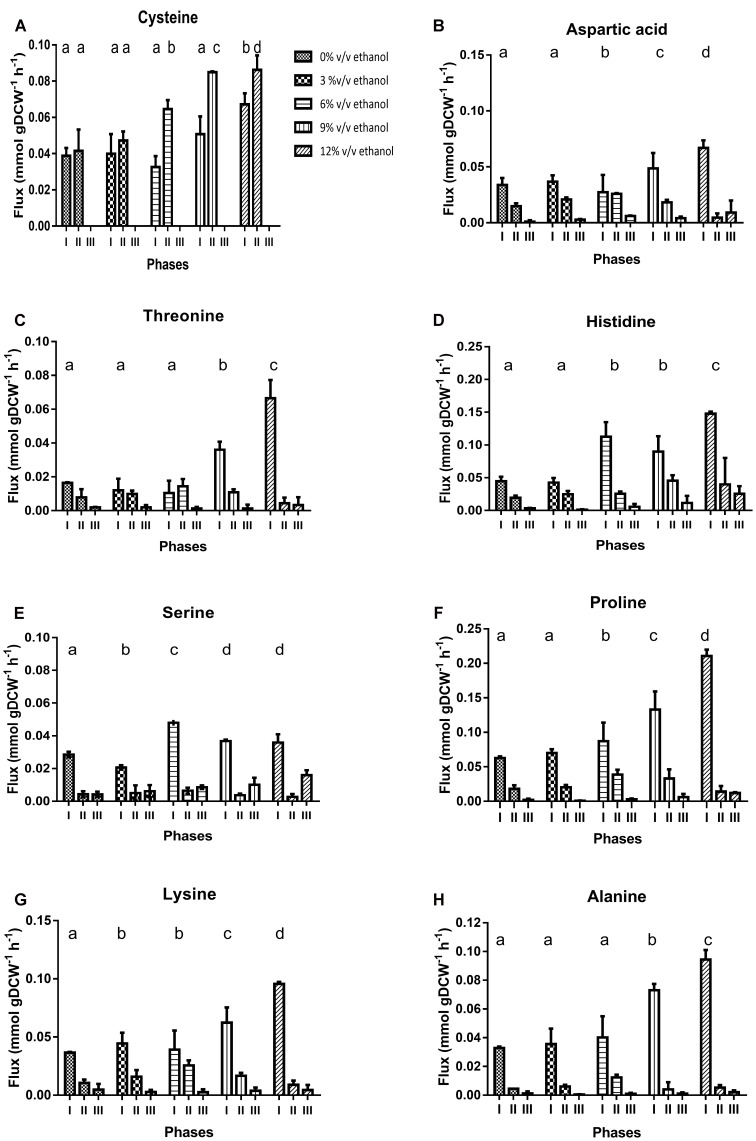
Specific consumption rates of amino acids during cultivation of *O. oeni* PSU-1 under increasing ethanol contents. **(A)** Cysteine. **(B)** Aspartic acid. **(C)** Threonine. **(D)** Histidine. **(E)** Serine. **(F)** Proline. **(G)** Lysine. **(H)** Alanine. Statistical analysis was only performed for phase I and shared letters indicate no significant difference (Mood test, *p* < 0.05).

All the amino acids were metabolized during the cultivation of *O. oeni* in MaxOeno culture medium at increasing concentrations of ethanol; however, none was totally consumed (**Table 2**). Histidine, cysteine, lysine and aspartic acid were the most consumed amino acids. The highest specific consumption rates were determined for histidine and proline (**Figures 5D,F**) and their rates increased concomitantly with increasing ethanol contents in culture medium.

**Table 2:** Amino acid requirements by *O. oeni* PSU-1 cultured in medium with different ethanol content.

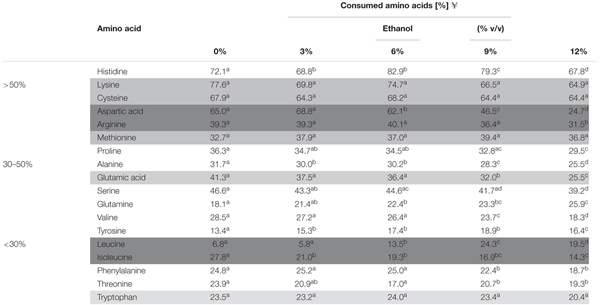

*^¥^All assays were performed in triplicate and all coefficients of variation (CVs) were <10%. Shared superscript letters (a, b, c) in the same row indicate no significant difference (Mood test, *p* < 0.05). Consumption of amino acids highlighted in light gray are consumed independently of ethanol content present in the culture medium. Consumption of amino acids highlighted in dark gray are consumed dependent of ethanol content present in the culture medium.*

Amino acids were mostly consumed (almost 90%), during phase I, with the exception of cysteine, which was consumed during both, phases I and II (**Figure 5** and **Supplementary Figure S3**). Interestingly, specific consumption rate of cysteine was the only one that was higher for phase II than for phase I, even increasing with ethanol content (**Figure 5A**).

Threonine was consumed faster in cultures with 9 and 12% ethanol; and serine was consumed faster in cultures with 6% ethanol (**Figures 5C,E**).

Besides, histidine and lysine consumption increased with ethanol. It is noteworthy that histidine was the most consumed amino acid in the cultures with 12% ethanol, reaching 18 mmol gDCW^-1^.

Cysteine, lysine, methionine, and tryptophan were up taken at similar amounts, whatever the ethanol concentration (**Table 2**). However, their specific consumption rates increased with ethanol content (**Figures 5A,G** and **Supplementary Figure S3**). Alanine and aspartic acid were also consumed faster in cultures with higher ethanol content (**Figures 5B,H**).

Finally, the consumption of aspartic acid, arginine, glutamic acid, and isoleucine decreased as ethanol content was incremented in the culture medium.

### Analysis of Intracellular Fluxes

#### Non-growth Associated Maintenance *(NGAM)*

Flux balance analysis was carried out in iSM454 model to predict NGAM for each experimental data set. Thereby, a range of possible NGAM values was tested and biomass formation rate was predicted. The NGAM value that allowed the minimal biomass prediction error was selected for each data set. Biomass was predicted correctly, with an average biomass prediction error of 0.05%.

**FIGURE 6:**
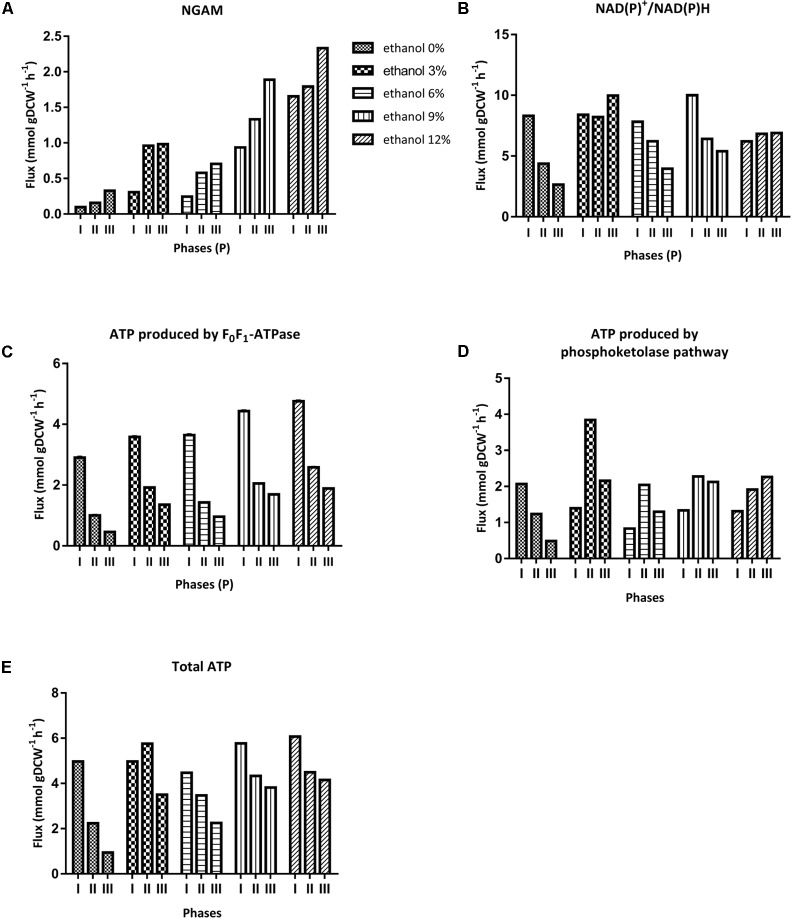
Non-growth associated maintenance (NGAM) and *in silico* determined specific production rates of key metabolites of *O. oeni.* **(A)** NGAM, **(B)** NAD(P)^+^/NAD(P)H, **(C)** ATP produced by F_0_F_1_-ATPase, **(D)** ATP produced by phosphoketolase pathway, **(E)** Total ATP, (i.e.) ATP produced by both F_0_F_1_-ATPase and phosphoketolase pathways.

Estimated NGAM values significantly increased in all phases, although differently, depending on the ethanol content of the culture medium. In phase I, NGAM increased threefold in cultures with 3 and 6% ethanol; and 10- and 17-fold in cultures with 9 and 12% ethanol. In phase II, it sequentially increased from 6 to 11 fold in cultures from 3 to 12% ethanol, respectively. In phase III, a maximum NGAM increase of sevenfold was achieved for cultures with 12% ethanol content. Whatever the case, at any ethanol level, NGAM was the lowest for phase I and the largest for phase III (**Figure 6A**).

#### NGAM Sensitivity Analysis

Sensitivity of NGAM values toward the experimental data was evaluated by varying each constrained flux independently in 1% of its value, and then repeating NGAM prediction. NGAM prediction variation was assessed by calculating the relative prediction error (in %), and the highest prediction error was taken as representative of the NGAM’s sensitivity of the corresponding data set (**Supplementary File S2**). Phase I was the most sensitive phase at every ethanol level, reaching the highest NGAM prediction variation in the absence of ethanol (130%). The largest variations were obtained when fructose and mannitol fluxes were varied.

#### Energetic Requirements

The model predicted that ATP requirements increase with ethanol content at each phase. Higher ATP requirement is predicted during phase I, whatever the ethanol content.

ATP is synthesized either by F_0_F_1_-ATPase or phosphoketolase pathway. In phase I, the maximum specific production rate of ATP (rATP) produced by F_0_F_1_-ATPase was higher than ATP produced through the phosphoketolase pathway. In cultures with 0 to 12% ethanol, fluxes through F_0_F_1_-ATPase increased from 2.9 to 4.8 mmol gDCW^-1^ h^-1^, respectively; and fluxes through the phosphoketolase pathway decreased from 2.1 to 1.3 mmol gDCW^-1^ h^-1^, for the same cultures (**Figures 6C,D**).

In phase II, r_ATP_ occurring through F_0_F_1_-ATPase decreased by 50% in all cultures. On the contrary, r_ATP_ through the phosphoketolase pathway increased in all cultures with ethanol content. Finally, during phase III, r_ATP_ by F_0_F_1_-ATPase continues to decrease – by another 50% compared to phase II; and r_ATP_ through phosphoketolase pathway also decreased in cultures containing less than 6% v/v of ethanol content but remained constant in cultures with higher ethanol contents (**Figures 6C,D**).

Interestingly, during phase I, the ATP flux through F_0_F_1-_ATPase increased from 58% to almost 80% of total ATP flux in cultures without ethanol and cultures with ethanol content, respectively. Besides, during phases II and III there was a preference for synthesis by the phosphoketolase pathway; on average, 56% of ATP was produced from this pathway (**Figures 6C–E**).

#### Cofactor Requirements

We determined the impact of ethanol on the cofactor usage by analyzing the NAD(P)H flux during growth (**Figure 6B**). The reactions that produce NAD(P)H involve the following enzymes: malate dehydrogenase, glyceraldehyde-3P dehydrogenase, threonine dehydrogenase, NADH quinone reductase, NAD(P)^+^ transhydrogenase and the pathway of methylglyoxal degradation for NADH formation; and glucose-6P dehydrogenase, phosphogluconate dehydrogenase and GMP reductase for NADPH formation. A clear trend of the use of cofactors in relation to the presence of ethanol in the culture medium could not be found (**Figure 6B**).

#### Elementary Flux Mode Analysis

As the MaxOeno medium contains several substrates, a EFM was carried out to determine possible substrate-product relationships between the substrates present in the medium and the possible products, including but not limited to products measured experimentally (**Table 3**). Results showed that serine, threonine and cysteine can be used for diacetyl formation, as well as citrate, which can also be a precursor for acetate production. L-malate can be directed into either D- or L-lactate, and therefore it is the only substrate that can be used for L-lactate synthesis. Both glucose and fructose are able to generate all products but L-lactate.

**Table 3:** Determination of possible substrate-product relationships by EFMA.

	D-mannitol	D-lactate	L-lactate	Diacetyl	Ethanol	Acetate	Erythritol
D-fructose	36	15	0	15	33	64	33
D-glucose	157	67	0	61	126	233	145
Citrate	0	0	0	2	0	2	0
L-malate	0	2	2	0	0	0	0
L-cysteine	0	0	0	3	0	0	0
L-serine	0	0	0	3	0	0	0
L-threonine	0	0	0	3	0	0	0


*EFMA was carried out in a reduced version of the iSM454 model. Position i, j indicates the quantity of pathways that included consumption of only substrate i (other substrates in zero), and generation of the production j.*

#### Random Sampling

100.000 flux distributions were computed using optGpSampler for each ethanol level and each phase. In each of the 15 conditions, the network was constrained using uptake and production rates as well as sink reactions for simulating accumulations. We used these flux distributions to study the flexibility of the network in each condition.

Means and standard deviations were calculated for 40 reactions related to phosphoketolase pathway. Overall, standard deviations were higher in phase I and smaller in phase III for all ethanol levels. Thus, the metabolic network is more flexible in phase I than in phase III. This is expected from a biological perspective as at the beginning of the fermentation there are more nutrients in the extracellular space – and with a higher concentration – than in later phases. Therefore, a higher number of reactions are expected to be available in order to catabolize these sugars. In contrast, at the end of the fermentation, citrate and malic acid are already depleted and other nutrients are almost exhausted resulting in a tighter metabolic network.

#### Metabolic Flux Distribution

**FIGURE 7:**
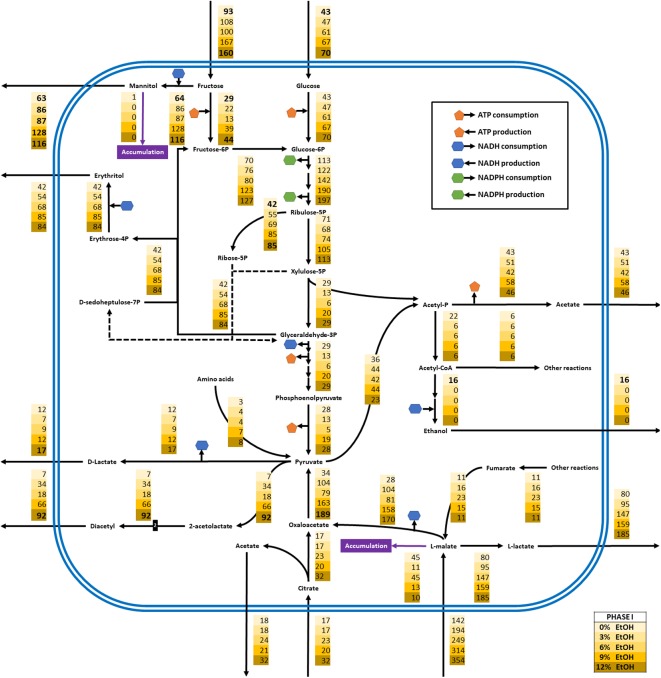
Metabolic flux redistribution of the central carbon metabolic pathways of *O. oeni* PSU-1 upon cultivation in a culture medium without and with 3, 6, 9, and 12% (yellow boxes, from top to bottom) ethanol concentration, during growth phase I. Polygons with colors orange, blue and green indicate consumption (or production) of ATP, NADH and NADPH, respectively.

Distribution of carbon intracellular fluxes was analyzed for all the growth phases, considering the major central metabolic pathways, which includes phosphoketolase pathway, fructose reduction, citrate degradation and MLF. FBA showed that a significant redistribution of intracellular fluxes occurred in *O. oeni* in response to ethanol content (**Figure 7** and **Supplementary Figures S4**, **S5**). Fluxes were normalized over specific growth rates.

The model showed that fructose, the sugar that was most consumed, was mainly used – around 70% of the total – for mannitol biosynthesis during phase I; however, in later phases, fructose was redirected to glucose-6P synthesis, and then into the phosphoketolase pathway (**Figure 7** and **Supplementary Figures S4**, **S5**).

Erythritol biosynthesis arises from ribulose-5P and glyceraldehyde-3P in almost all cases (**Figure 7** and **Supplementary Figures S4**, **S5**). It was highest during phase I, and increased with ethanol content, from 42 to 84 mmol gDCW^-1^ in cultures without and with 12% ethanol, respectively.

The model also predicted diacetyl synthesis. This specific production rates incremented with ethanol content and thus were highest at 12% ethanol in all phases. This compound is produced from pyruvate.

Interestingly, pyruvate production increased in phase II, and even more in phase III by the following routes: amino acid degradation, citrate degradation, and L-malate conversion into oxaloacetate (**Figure 7** and **Supplementary Figures S4**, **S5**). All of these fluxes also incremented as ethanol increased in the culture medium (**Figure 7** and **Supplementary Figures S4**, **S5**).

## Discussion

In this work, we determined the effect of ethanol on nutritional and energetic requirements of *O. oeni* to ensure its growth. For this end, we cultivated *O. oeni* PSU-1 strain in a wine-like, defined culture medium spiked with 0, 3, 6, 9, or 12% v/v ethanol. Moreover, we took advantage of our recently constructed genome-scale metabolic model (Mendoza et al., 2017) that allowed assessing the redistribution of the intracellular metabolic fluxes and the energetic factors at increasing ethanol concentrations.

As expected, cell growth was closely related (*r*^2^ = 0.98) to ethanol content. Specific growth rate and maximal biomass content decreased progressively as ethanol concentration in the medium increased. Moreover, both decreased during the time course of the batch cultivation. In general, specific growth rates and biomass production were larger during phase I, which was coincident with the highest metabolic activity of *O. oeni* observed during this phase, for any culture condition.

Changes in ethanol level strongly impacted *O. oeni’s* requirements of NGAM, NAD(P)^+^/NAD(P)H cofactors and energy, reflected in higher production of ATP by F_0_F_1_-ATPase.

Energetic demand for NGAM dramatically increased in *O. oeni* cells cultured under alcoholic stressful conditions. Indeed, cultures containing 9 and 12% ethanol required 10 and 17 times more NGAM during phase I, respectively, than cultures without ethanol. The latter indicates that the ATP produced was principally used to cope with cell maintenance resulting from this stress. This agrees with our previous work, where the genome –scale metabolic model (GSMM) indicated that NGAM was 30-fold higher in cultures with 12% ethanol than in cultures without ethanol (Mendoza et al., 2017). Notably, the model predicted that the cells cultivated with 6% ethanol would require a lower NGAM than those grown at 3% ethanol. This agrees with Cavin et al. (1998) that reported that low concentrations of ethanol activate bacterial growth, (i.e.) ethanol improves exchanges between the cell and the external medium. In addition, significant cellular changes normally occur when at least 8% ethanol is present in the medium, coinciding with changes in membrane lipid composition (Teixeira et al., 2002; Da Silveira et al., 2003; Grandvalet et al., 2008).

F_0_F_1_-ATPase is the favorite route employed for the synthesis of ATP during phase I. Indeed, even in the absence of ethanol in the culture, 58% of the total ATP is generated by this route. Moreover, to overcome the strong ATP demand for NGAM at higher ethanol concentrations, the model predicts that this pathway produces almost 80% of the required ATP. As F_0_F_1_-ATPase produces ATP at the expense of proton translocation to the inside of the cell, this indicates a higher requirement for proton extrusion. In this regard, the malolactic reaction is usually considered the major pathway for proton extrusion and ATP production in LAB (Tourdot-Maréchal et al., 1999; van de Guchte et al., 2002). In this process, one mole of malic acid is consumed and generates equivalent amounts of L-lactate and CO_2_; and, because of pKa differences between the substrate and the products, a proton is consumed (Marty-Teysset et al., 1996; Konings, 2002; Augagneur et al., 2007). This positive effect is augmented by L-lactate transport, a symporter that extrudes protons (Salema et al., 1996). We found that malic acid was almost totally depleted during phase I in each condition, in concordance with the higher F_0_F_1_-ATPase flux predicted by the model. Moreover, in this phase, specific consumption rates of malic acid were the fastest in cultures with 9 and 12% ethanol, as well as specific production rates of L-lactate. The key role of malic acid for proton extrusion is confirmed by the model, as it predicts that 74–95% of the protons translocated by F_0_F_1_-ATPase are extruded by MLF and L-lactate transport in all the experimental conditions; and that the flux through this reaction increases concomitantly with the ethanol content in the medium, to overcome higher energetic requirements. This confirms that malolactic reaction is the main pathway for ATP synthesis in the presence of high ethanol content.

For reductive power regeneration, ethanol formation is the main pathway for reoxidation of NAD(P)^+^ in *O. oeni*, although at high metabolic rates this process becomes limiting (Richter et al., 2003); therefore, other external electron acceptors are used for NAD(P)^+^ reoxidation (Versari et al., 1999; Maicas et al., 2002; Richter et al., 2003). Several studies have reported that one of the main limitations in NAD(P)^+^ regeneration through the ethanol biosynthetic pathway is the deficiency of D-pantothenate in the culture medium, an essential precursor for HSCoA in *O. oeni* (Richter et al., 2001; Terrade and Mira de Orduña, 2009). The latter is the cofactor of acetaldehyde dehydrogenase (Garvie, 1967). Nevertheless, the MaxOeno culture medium employed in this work contains enough D-panthotenate to allow optimal growth of *O. oeni* and ethanol pathway activation. Another cause could be the limited availability of HSCoA due to its preferential use for fatty acid production, to overcome the damage of cell membrane that could result from ethanol.

When ethanol production pathway is non-functional, a lack of reduced cofactors occurs. Our results show that *O. oeni* PSU-1 uses fructose, glucose, and citrate as electron acceptors for cofactors reduction. In particular, the specific production rates of mannitol and erythritol incremented as ethanol concentrations increased during phase I. However, fructose, which is the main precursor of mannitol and erythritol, showed similar specific consumption rates during phase I, whatever the culture conditions; and glucose, that can also be used in erythritol formation, displayed slower consumption rates at higher ethanol concentrations. This indicates that there is a preferential consumption of carbon sources toward mannitol and erythritol formation at higher ethanol concentrations rather than biomass formation, as a result of higher cofactor requirements. Indeed, the model predicts that cofactor regeneration due to mannitol and erythritol formation countervail for almost 50% of the cofactors used for sugar catabolism in conditions without ethanol, and for 51–57% of this usage in ethanol-containing cultures.

Citric acid consumption is also related with cofactor regeneration. Consumption of this chemical compound is used for pyruvate formation, which is then used for either production of D-lactate or diacetyl. D-lactate is used for NAD(P)^+^ regeneration, while diacetyl is related with consumption of intracellular protons and thus increase of internal, as well as external pH, as diacetyl is less acidic than citric acid (Saguir and Manca de Nadra, 1996; Versari et al., 1999). Citric acid was mainly consumed during phase I at similar specific consumption rates, whatever the ethanol content. During this phase, D-lactate formation showed a different behavior: the highest flux toward D-lactate synthesis – which arises from both, citrate degradation and phosphoketolase pathway – was observed when ethanol was absent in the medium, where 72% of the consumed citrate was directed toward the synthesis of lactate, instead of the 38 and 58% observed at 9 and 12% v/v ethanol, respectively. On the contrary, the model predicted higher diacetyl production fluxes when ethanol was higher in the medium. As citric acid consumption was similar in all conditions, this indicates that, at high ethanol concentrations, citric acid consumption was not sufficient to supply all the pyruvate required for diacetyl synthesis. Indeed, in the absence of ethanol, 44% of the citrate consumed was used for diacetyl formation; however, at 9 and 12% ethanol, there were 3.2 and 2.9 moles of diacetyl produced per mole of consumed citrate. This clearly indicates that pyruvate was redirected to the formation of this compound. For this end, the required pyruvate is produced from L-malate, as at increasing ethanol conditions the flux through malic enzyme, which uses L-malic acid for oxaloacetate synthesis, increases. Thus, a 3.7-fold higher synthesis of oxaloacetate was achieved through this route in cultures containing 12% ethanol, as compared to those without ethanol.

Thus, citric acid was mainly used for internal ionic balance through diacetyl production under ethanolic conditions; and not for NAD(P)^+^ cofactor regeneration. In LAB, diacetyl formation requires intracellular protons, resulting in an increase in the internal pH. In addition, its extrusion increases external pH because it is less acidic than citric acid. In general, D-lactate production is privileged with regards to diacetyl, probably to allow the cells to obtain NAD^+^; however, when lactate dehydrogenase (LDH) function is reduced, pyruvate accumulates, and diacetyl is produced (García-Quintáns et al., 1998; Tsakalidou and Papadimitriou, 2011). Thus, high ethanol content could limit the function of LDH, allowing diacetyl production, as demonstrated here. Another possible cause is that higher diacetyl formation results from the larger energetic requirements caused by higher NGAM requirements. As F_0_F_1_-ATPase generates ATP by translocating protons to the inside of the cell, higher ethanol concentrations imply a higher necessity to extrude these protons by alternative pathways. In fact, at the highest ethanol concentrations, diacetyl generation consumes 20 and 25%, respectively, of the protons introduced into the cell by F_0_F_1_-ATPase; opposite to only 5% for cells grown in the absence of ethanol. This shows that the increment of diacetyl formation and the resulting proton consumption in this reaction allows to increment the proton gradient. This gradient can then be used to produce, through F_0_F_1_-ATPase, the energetic requirements needed to overcome the challenging environment of this elevated ethanol concentration in the medium. From a biological perspective, proton consumption can also be used to compensate for the proton influx caused by the higher permeability of the plasmatic membrane at high ethanol concentrations.

Other authors have cultured *O. oeni* PSU-1 in the presence of 12% of ethanol, showing that genes related with malate and citrate consumption were up-regulated while genes related with fructose consumption were down-regulated, which was associated to mechanisms of ethanol resistance (Margalef-Catalá et al., 2016). We observed that malate and citrate were almost totally consumed during the first 48 h of culture (phase I), suggesting that both substrates were critical for *O. oeni* survival; although we did not find a clear relation between ethanol content and citrate consumption, we did observe an increment in fluxes related with citrate consumption. Moreover, we found that fructose and glucose were consumed faster during phase I, without any inhibition.

Additionally, the specific consumption rate of cysteine, one of the most consumed amino acids, increased with ethanol content in the medium. Genomic studies of *O. oeni* PSU-1 reported that this strain is unable to synthesize cysteine, because sulfur cannot be transported inside of the cell (Garvie, 1967; Mills et al., 2005). Cysteine can be used as a source for pyruvate formation, together with serine and threonine. The model predicted a 2.7-fold increase in pyruvate formation from these amino acids at 12% ethanol than in cultures without ethanol. Additionally, cysteine can be used in reactions of CoA synthesis, where this amino acid is added to D-4-P-phantothenate generating R-4-P-phantothenosyl-L-cysteine, and *O. oeni* does have the genes to this synthesize (Mills et al., 2005). CoA functions as an acyl group carrier and carbonyl activating group in numerous reactions central to cellular metabolism and provides the 40-phosphopantetheine prosthetic group incorporated by carrier proteins that play key roles in fatty acid and non-ribosomal peptides biosynthesis (Spry et al., 2008).

## Conclusion

We found that under ethanol stress conditions, *O. oeni* favors anabolic reactions related with cell reconstruction pathways and/or production of stress protectors; consequently, the requirements of NAD(P)^+^, NGAM and ATP increase with ethanol content, unrelated with biomass increment.

Finally, in this work we were able to integrate in the model specific consumption/production rates and specific growth rates for each of the determined growth phases, and thus, the model was able to represent the different phenotypes of *O. oeni* in each of the growth phases. To the best of our knowledge, this is the first report where experimental data from the entire exponential curve has been integrated to the model (Mendoza et al., 2017). Even if GEMs and FBA are usually applied to data obtained from steady state experiments, these strategies have been previously used to model data from batch growth, based on the assumption of a pseudo-steady state of the cellular metabolism (Teusink et al., 2005; Pastink et al., 2009; Pereira et al., 2016). The approach developed in this work allowed to characterize the physiological changes that occur in the metabolism of *O. oeni* PSU-1 during its growth in a culture medium containing various carbon sources, as the wine-like medium MaxOeno. The results highlight the flexibility of the metabolism of this bacterium, which could not have been carried out with a canonic approach (Mendoza et al., 2017); for example, we previously reported that at high ethanol concentrations, production of ATP was preferentially carried out through the phosphoketolase pathway. In the present study, where dynamic changes were assessed, a similar result was found for phases II and III; however, the opposite was observed for phase I, a result that was previously lost due to the canonical analysis; the latter stresses the critical role of organic acids for ATP synthesis and, thus, for survival and adaptation in a medium with high ethanol concentrations. Furthermore, we were able to identify and include in our simulations the internal accumulation of compounds such as mannitol, which had an impact in the pathways used on each phase for cofactor regeneration.

Several perspectives derived from this study can be foreseen. For example, a similar approach can be employed to tackle the metabolic response to acidic conditions in this bacterium, which, together with alcohol and SO_2_ stresses, are the most relevant conditions that affect the growth of *O. oeni.* For instance, Liu et al. (2017) recently showed, using RNA-seq, that in *O. oeni* SD-2a strain grown at pH of 4.8 and pH 3.0, several genes related with the metabolism of amino acids, carbohydrates, membrane transport and energy metabolism were differentially expressed. Therefore, the resulting quantitative transcriptomics can be incorporated into the iSM454 metabolic model as restrictions and, together with experimental data, could allow to simulate the redistribution of metabolic fluxes resulting from this environmental perturbation.

Moreover, important efforts have been made to identify the genetic characteristics of different communities of *O. oeni* from different geographical regions, which is particularly relevant to understand the relationship between the wines of a certain geographical location and what has been termed as the “terroir” (e.g., Capozzi et al., 2014). The characterization of these genomes is particularly relevant to determine different aspects of safety, tolerance to the harmful wine conditions and its contribution of the sensory quality of the wine. Thus, an approach like the one proposed in this paper, considering the genomic differences at the strain-specific level, would allow modeling the metabolic behavior of strains that are of interest for wines within a geographical location and to understand the physiological conditions in which a metabolic shift occurs. For example, the metabolic model used here is based on the PSU-1 strain, which does not produce biogenic amines, since the biosynthetic pathways of these compounds have not been found in the genome of *O. oeni* PSU-1. However, it is known that most *O. oeni* strains produce these deleterious compounds (Lucas et al., 2008; López et al., 2009). Therefore, the inclusion of the related biosynthetic pathway in the *O. oeni*’s GEM to simulate their biosynthesis under different cultural and environmental conditions, to understand and minimize their production will be particularly promising.

Finally, and in accordance with the above, determination of new ecotypes will permit to define new nutritional formulations that might be relevant for the wine industry, such as tolerance to alcohol, or resistance to SO_2_ and pH, as well as efficient consumption of malic acid. For example a recent study carried out in 16 wineries from different Chilean valleys showed that autochthonous strains present a unique number of genes with respect to commercial strains. Moreover, some of these strains do not contain some genes related to off-flavors, such as phenolic decarboxylase (Romero et al., 2018). Thus, physiological studies complemented with the GEM of several indigenous *O. oeni* strains might unravel new organoleptic patterns, as well as environmental responses, which complement the current knowledge regarding the differences at genomic level and, eventually, discover new starter cultures.

## Author Contributions

EA designed the research and provided guidance throughout the investigation. AC wrote the paper, designed, performed, and coordinated the batch experiments as well as processed the HPLC data. AC and GG designed and evaluated the defined culture medium MaxOeno. MR, SM, and PC developed the modeling framework. MR developed the extended model and executed the modeling simulations and analyses. SM supervised these analyses. PC designed the figures of metabolic flows and contributed to biochemical interpretation of metabolic reaction added to the model. All the authors read, corrected, and approved the final manuscript.

## Conflict of Interest Statement

The authors declare that the research was conducted in the absence of any commercial or financial relationships that could be construed as a potential conflict of interest.

## Supplementary Material

**FIGURE S1 |** Time course of fructose consumption and metabolite production by *O. oeni* PSU-1, cultured at different ethanol concentrations. **(A)** Fructose consumption. **(B)** Glucose consumption. **(C)** Mannitol production. **(D)** Erythritol production and **(E)** D-lactate production.

**FIGURE S2 |** Time course of some of substrates and metabolites produced mainly by *O. oeni* PSU-1, cultured at different ethanol concentrations. **(A)** L-malate consumption. **(B)** L-lactate production. **(C)** Citrate consumption and **(D)** acetate production.

**FIGURE S3 |** Specific amino acids consumption rate by *O. oeni* PSU-1, during cultivation under increasing ethanol contents. **(A)** Glutamic acid. **(B)** Glutamine. **(C)** Valine. **(D)** Methionine. **(E)** Arginine. **(F)** Tyrosine. **(G)** Tryptophan. **(H)** Leucine. **(I)** Isoleucine and **(J)** Phenylalanine. Statistical analysis only was performed in phase I and shared letters indicate no significant difference (Mood test, *p* < 0.05).

**FIGURE S4 |** Metabolic flux redistribution of the central carbon metabolic pathways of *O. oeni* PSU-1 upon cultivation in a culture medium without and with 3, 6, 9, and 12% (green boxes, from top to bottom) ethanol concentration, during growth phase II.

**FIGURE S5 |** Metabolic flux redistribution of the central carbon metabolic pathways of *O. oeni* PSU-1 upon cultivation in a culture medium without and with 3, 6, 9, and 12% (orange boxes, from top to bottom) ethanol concentration, during growth phase III.

**FILE S1 |** Reactions contained in the reduced version of the iSM454 model.

**FILE S2 |** Sensitivity analysis of NGAM estimation with respect to the fixed specific consumption/production rates of compounds during growth of *O.oeni* PSU-1 at different ethanol concentrations in maxOeno medium.
