# Supplementary figures and images for: Mapping the Physiological Response of Oenococcus oeni to Ethanol Stress Using an Extended Genome-Scale Metabolic Model
Source: Front Microbiol. 2018 Mar 1;9:291. doi: 10.3389/fmicb.2018.00291 (PMC5838312; doi:10.3389/fmicb.2018.00291)

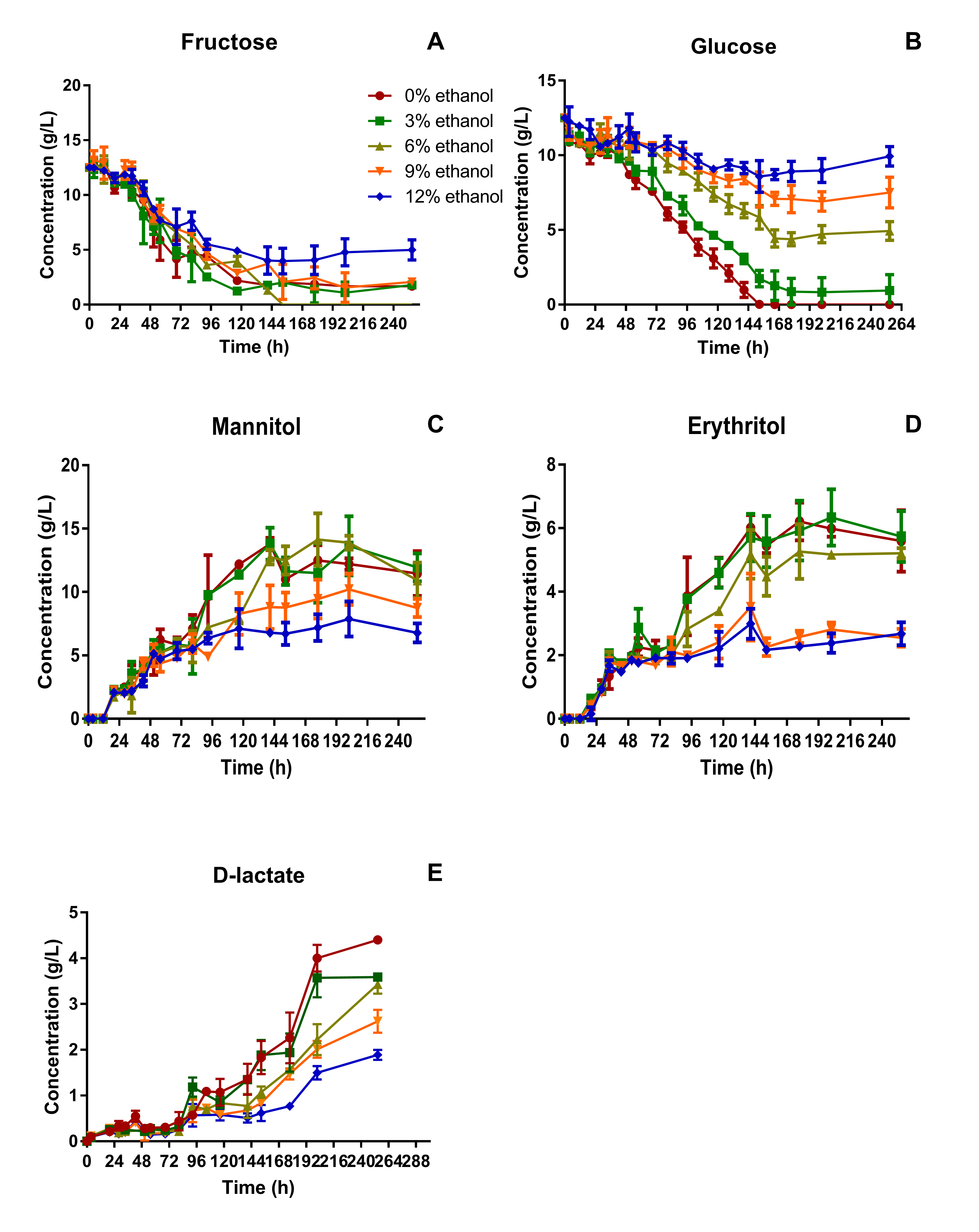

Supplement: Supplementary file 1 [file Image_1.TIF]

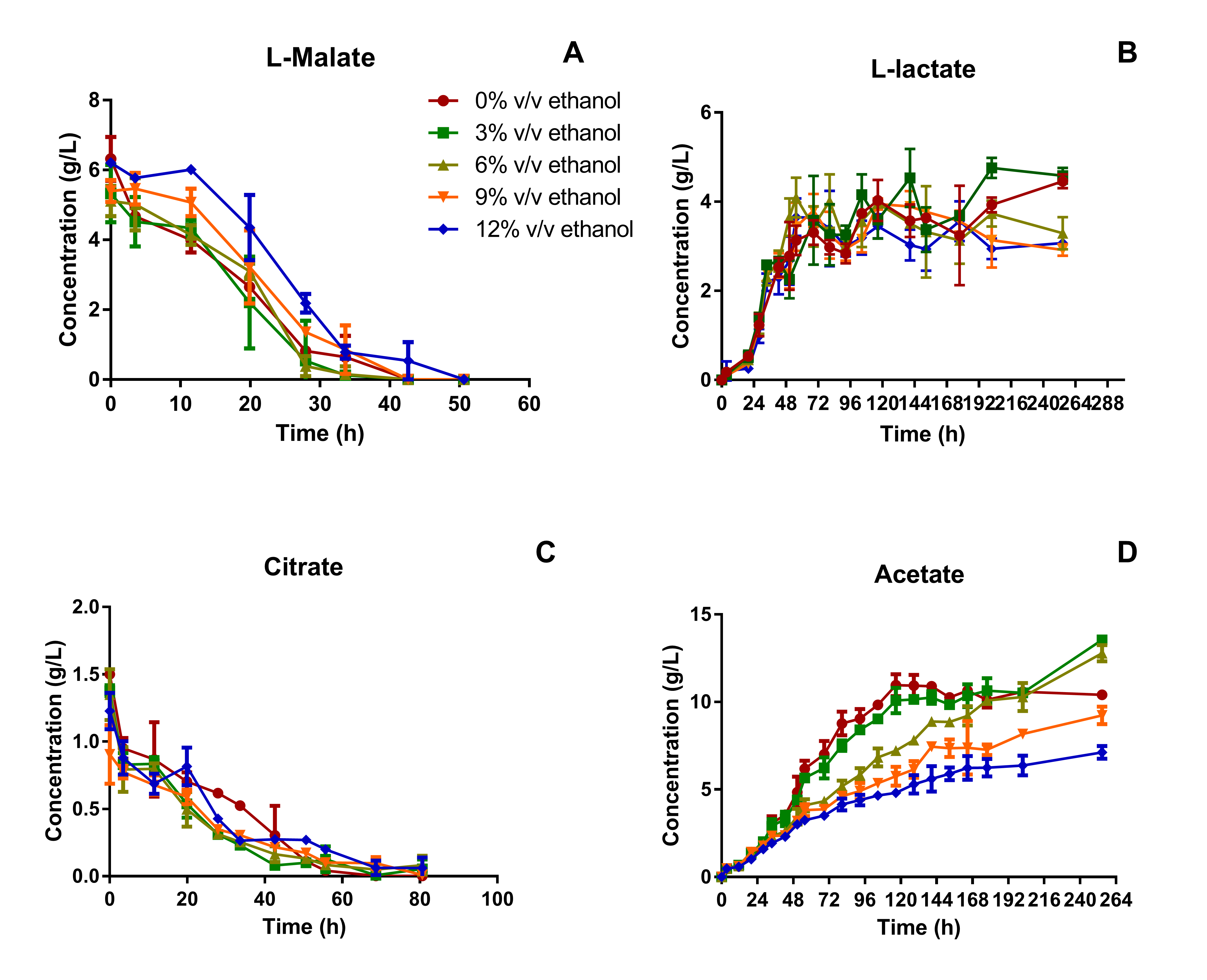

Supplement: Supplementary file 2 [file Image_2.TIF]

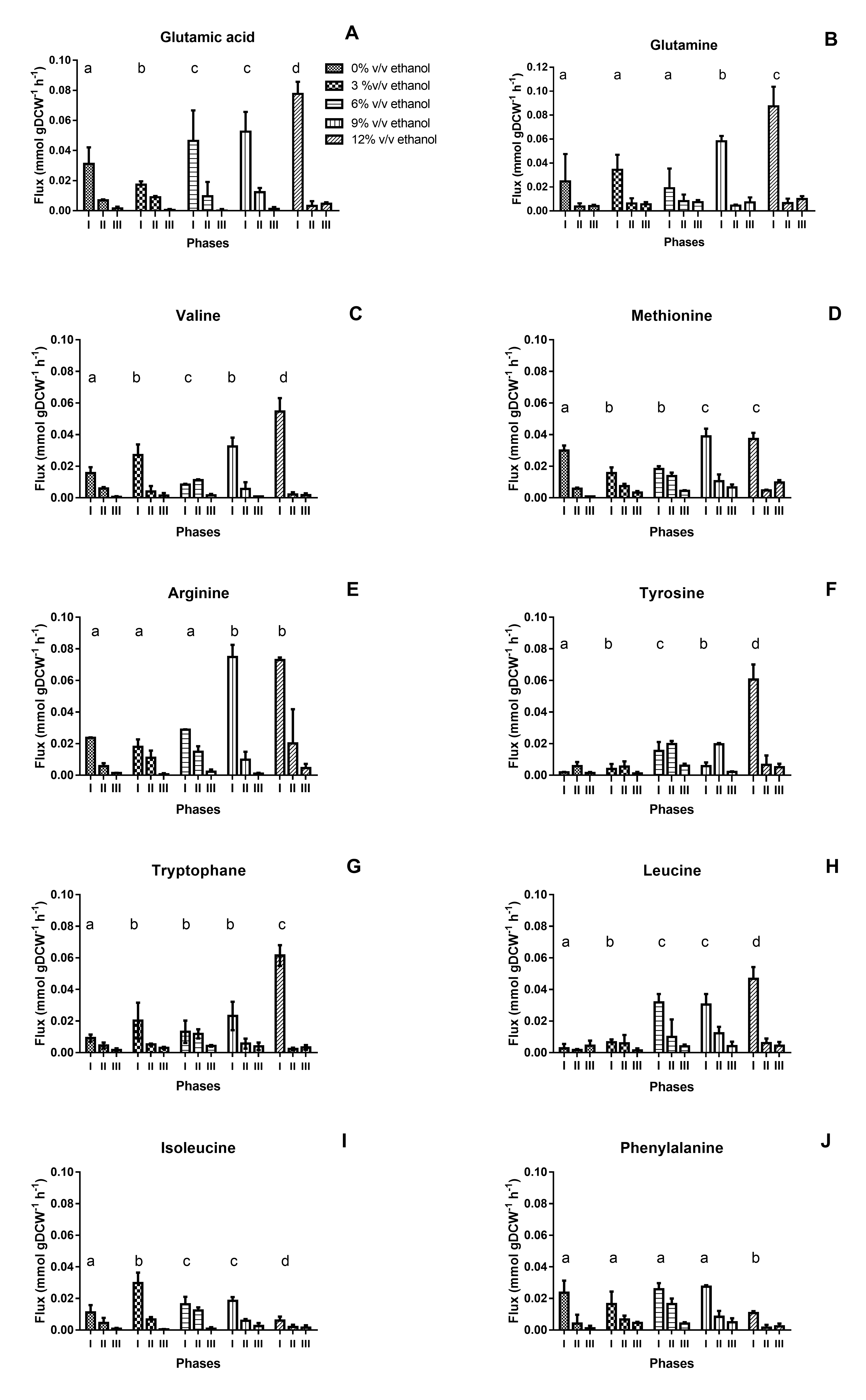

Supplement: Supplementary file 3 [file Image_3.TIF]
