# Supplementary material for: Mapping the Physiological Response of Oenococcus oeni to Ethanol Stress Using an Extended Genome-Scale Metabolic Model
Source: Front Microbiol. 2018 Mar 1;9:291. doi: 10.3389/fmicb.2018.00291 (PMC5838312; doi:10.3389/fmicb.2018.00291)
Supplement: Supplementary file 4 [file Image_4.pdf]

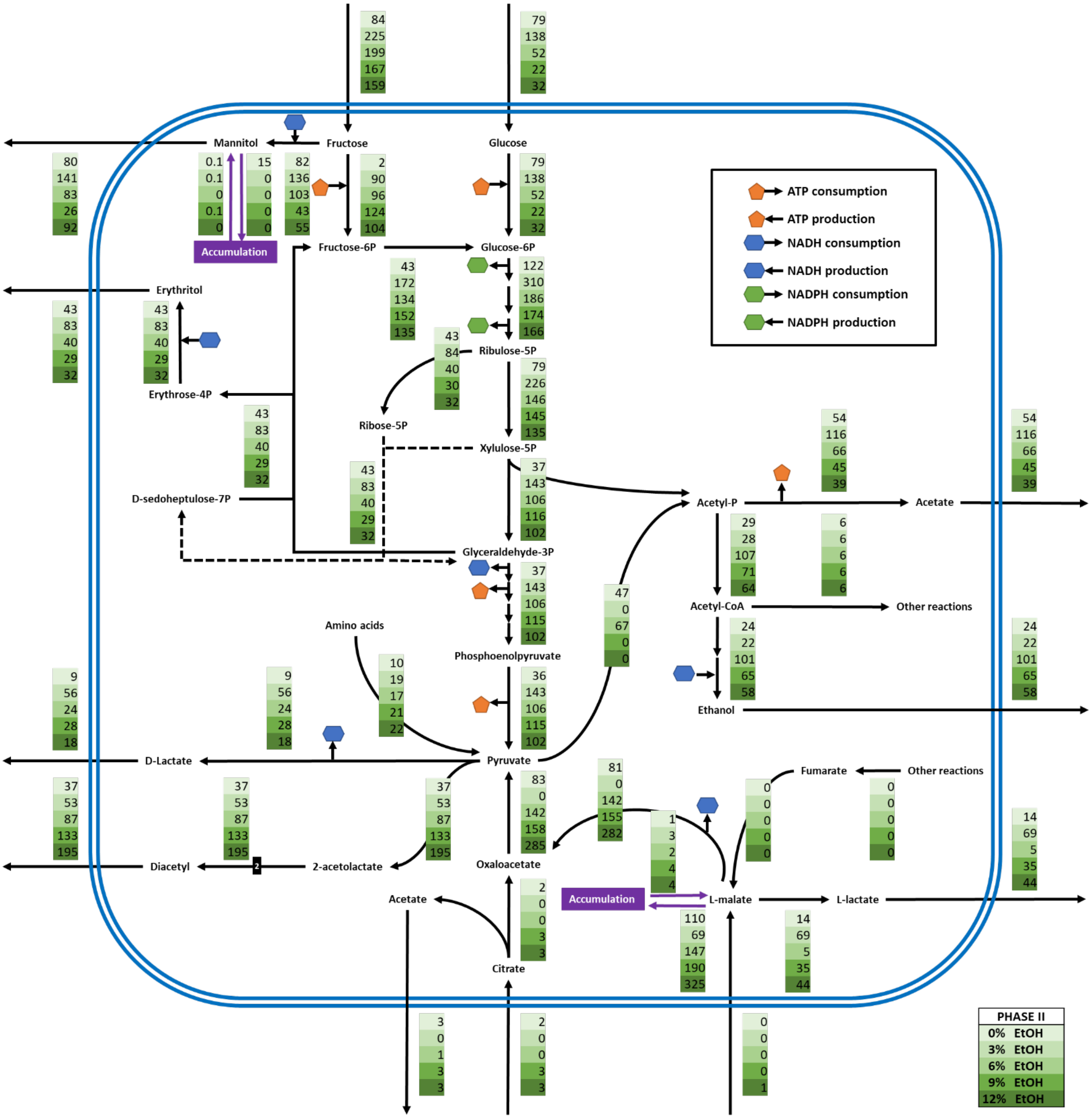

Supplementary Figure S4. Metabolic flux redistribution of the central carbon metabolic pathways of *O. oeni* PSU-1 upon cultivation in a culture medium without and with 3, 6, 9 and 12% (green boxes, from top to bottom) ethanol concentration, during growth phase II.
