# Supplementary material for: Mapping the Physiological Response of Oenococcus oeni to Ethanol Stress Using an Extended Genome-Scale Metabolic Model
Source: Front Microbiol. 2018 Mar 1;9:291. doi: 10.3389/fmicb.2018.00291 (PMC5838312; doi:10.3389/fmicb.2018.00291)
Supplement: Supplementary file 6 [file Data_Sheet_1.pdf]

Reactions contained in the reduced version of the iSM454 model

The reduced version of the iSM454 model includes the following reactions from the original file:

'_2PGADEHYDRAT_RXN'
'PHOSACETYLTRANS_RXN'
'ACETATEKIN_RXN'
'PEPDEPHOS_RXN'
'GLUCOKIN_RXN'
'PHOSGLYPHOS_RXN'
'PGLUCISOM_RXN'
'GAPOXNPHOSPHN_RXN'
'_3PGAREARR_RXN'
'GLU6PDEHYDROG_RXN'
'PHOSPHOKETOLASE_RXN'
'RXN66_3'
'ALCOHOL_DEHYDROG_RXN'
'DLACTDEHYDROGNADRXN'
'ACETALD_DEHYDROG_RXN'
'_6PGLUCONOLACT_RXN'
'ALDOSE_1_EPIMERASE_RXN'
'RXN_9952'
'ACETOLACTSYN_RXN'
'ACETOLACTATE_DECARBOXYLASE_RXN'
'RXN_11036'
'RR_BUTANEDIOL_DEHYDROGENASE_RXN'
'RXN_6081'
'CITLY_RXN'
'OXALODECARB_RXN'
'Mannitol_RXN'
'ERITHRITIOL_RXN'
'PHOSPHOKETOLASE2_RXN'
'_1_2_3_3_RXN'
'MALATE_DEH_RXN'
'FUMHYDR_RXN'
'PYRUVDEH_RXN'
'RXNK9E_163'
'TRANSALDOL-RXN'
'PRPPSYN_RXN'
'RIBULP3EPIM_RXN'
'_1TRANSKETO_RXN'
'RIB5PISOM_RXN'
'b_D_fructose_ex_'
'a_D_glucose_ex_'
'D_mannitol_ex_'
'citrate_ex_'
'_R__lactate_ex_'
'_S__lactate_ex_'
'_S__malate_ex_'
'butanediol_ex_'

'diacetyl_ex_'
'ethanol_ex_'
'ammonium_ex_'
'acetate_ex_'
'erythritol_ex_'
'CO2__ex_'
'oxygen_ex_'
'LCYSDESULF_RXN'
'CYSTATHIONINE_BETA_LYASE_RXN'
'METHYL_GLYOXAL_DEHYDROG_RXN'
'growth_rate'
'TRANS_ERITHRITOL_RXN'
'TRANS_RXNK9E_520'
'TRANS_RXNK9E_529'
'TRANS_RXNK9E_539'
'TRANS_RXNK9E_540'
'TRANS_RXNK9E_542'
'TRANS_RXNK9E_541'
'TRANS_RXNK9E_463'
'TRANS_RXNK9E_524'
'TRANSRXNK9E525'
'TRANS_RXNK9E_434'
'TRANS_RXNK9E_354'
'TRANS_RXNK9E_535'
'TRANS_RXNK9E_531'
'TRANS_RXNK9E_533'
'TRANS_RXNK9E_530'
'TRANS_RXNK9E_431'
'L_Cys_ex_'
'TRANS_RXNK9E_474'
'TRANS_RXNK9E_492'
'L_Ser_ex_'
'TRANS_RXNK9E_481'
'TRANS_RXNK9E_500'
'L_Thr_ex_'
'TRANS_RXNK9E_482'
'TRANS_RXNK9E_490'
'H2S_transport'
'H2S_exchange'
'water_ex_'
'TRANS_RXNK9E_519'
'ammonium_ex_'
'TRANS_RXNK9E_415'
'ATPSYN_RXN'
'RXNK9E_225'
'CYSTATHIONINE_BETA_SYNTHASE_RXN'
'AMACETOXID_RXN'
'THREOSPON_RXN'
'THREODEHYD_RXN'
'b_D_glucose_ex_'
'TRANS_RXNK9E_536'

'TRANS_RXNK9E_534'
'H__ex'
